# Radiological Assessment of the Indian Children with Congenital Sensorineural Hearing Loss

**DOI:** 10.1155/2014/808759

**Published:** 2014-07-14

**Authors:** Sangeet Kumar Agarwal, Satinder Singh, Samarjit Singh Ghuman, Shalabh Sharma, Asish Kr. Lahiri

**Affiliations:** ^1^Department of Otorhinolaryngology and Head, Neck Surgery, Sir Ganga Ram Hospital, New Delhi 110049, India; ^2^Department of Radiology, Sir Ganga Ram Hospital, New Delhi 110049, India

## Abstract

*Introduction*. Congenital sensorineural hearing loss is one of the most common birth defects with incidence of approximately 1 : 1000 live births. Imaging of cases of congenital sensorineural hearing loss is frequently performed in an attempt to determine the underlying pathology. There is a paucity of literature from India and for this reason we decided to conduct this study in Indian context to evaluate the various cochleovestibular bony and nerve anomalies by HRCT scan of temporal bone and MRI with 3D scan of inner ear in a tertiary care centre. *Material and Methods*. A total of 280 children with congenital deafness (158 males and 122 females), between January 2002 to June 2013 were included in the study and they were assessed radiologically by HRCT scan of temporal bone and MRI with 3D scan of inner ear. * Results*. In the present study we found various congenital anomalies of bony labyrinth and vestibulocochlear nerve. Out of 560 inner ears we found 78 anomalous inner ears. Out of these 78 inner ears 57 (73%) had cochlear anomaly, 68 (87.1%) had anomalous vestibule, 44 (56.4%) had abnormal vestibular aqueduct, 24 (30.7%) had anomalous IAC, and 23 (29.4%) had abnormal cochleovestibular nerves. *Conclusion*. In present study, we found lower incidences of congenital anomalies comparative to existing literature.

## 1. Introduction

Congenital sensorineural hearing loss is one of the most common birth defects with incidence of approximately 1 : 1000 live births [1]. Imaging of cases of congenital sensorineural hearing loss is frequently performed in an attempt to determine an underlying pathology. Both high resolution computed tomography scan (HRCT) of the temporal bone and magnetic resonance imaging scan (MRI) of the inner ear have been used in this set of patients with certain advantages and disadvantages of each. The HRCT scan reveals many types of bony inner ear malformations and MRI scan provides better visualization of the membranous labyrinth and the status of vestibulocochlear nerves. In such cases the most common CT scan abnormality is a dilated vestibular aqueduct (LVA) defined as measuring greater than 1.5 mm in diameter. This disorder may be unilateral or bilateral [1].

Bony inner ear malformations are fairly uncommon anomalies, representing approximately 20% of the cases of congenital sensorineural hearing loss. The remaining 80% of the cases of congenital malformations are membranous malformations in which bony architecture of the inner ear is normal and the pathology is at the cellular level. In the latter patient group, the result of radiological investigations of the inner ear falls within the normal limits [2].

Before the cochlear implant era, radiology of the temporal bone was not routinely done in prelingually deaf children. It was observed that a few cases of Michel deformity had been inadvertently fitted with hearing aids and rehabilitation was initiated. In order to avoid misfortunes like this, it is now common practice to obtain radiological evaluation of the temporal bone as soon as patient is diagnosed with severe to profound sensorineural hearing loss. Vestibulocochlear congenital anomalies may be classified as follows.

1. Michel deformity: there is complete absence of all cochlear and vestibular structures.
2. Cochlear aplasia: the cochlea is completely absent.
3. Common cavity deformity: there is a cystic cavity representing the cochlea and vestibule but without showing any differentiation into cochlea and vestibule.
4. Cochlear hypoplasia: the cochlea and vestibule are separate from each other but their dimensions are smaller than normal.
5. Incomplete partition type I: the cochlea is lacking the entire modiolus and cribriform area resulting in a cystic appearance. This is accompanied by a large cystic vestibule.
6. Incomplete partition type II (Mondini deformity): the cochlea consists of 1.5 turns in which the middle and apical turns coalesce to form a cystic apex, accompanied by a dilated vestibule and enlarged VA.
7. Vestibular malformations: They include Michel deformity, common cavity, absent vestibule, and dilated vestibule.
8. Semicircular canal malformations: They are absent, hypoplastic, or enlarged.
9. Internal auditory canal malformations: They are absent, narrow, or enlarged.

Radiology gives information regarding the type of malformation, additional pathologies in the middle ear and mastoid, and the presence or absence of the vestibulocochlear nerve. There has been a debate about which of the two modalities, HRCT or MRI, should be used in the preoperative evaluation of candidates undergoing cochlear implantation. HRCT scan of the temporal bone should be obtained in axial and coronal sections. This gives very good details of the temporal bone. Facial nerve abnormalities and the size of any defect between the internal auditory canal (IAC) and inner ear can be better evaluated on HRCT. MRI is important to diagnose the presence of nerves in the IAC and cochlear fluids [3].

Cochlear implants in patients with severe to profound sensorineural hearing loss have proved to be the method of choice for auditory rehabilitation. Accurate preoperative imaging is necessary for selection of candidates, identification of the more suitable ear for implantation, and selection of the appropriate device. The fluid filled cochlea and the cochlear nerve are the structures of highest interest for a successful surgery [4]. Radiological imaging plays a major role in cochlear implantation with regard to preoperative candidacy evaluation, intraoperative monitoring, and postoperative evaluation as well as research and experimental techniques. Imaging the auditory pathway of the implant candidate is necessary to screen for morphological conditions that will preclude or complicate the implantation process.

The selection of candidates for cochlear implantation requires consideration of a variety of clinical and radiographic factors. With the rising use of increasingly complex multichannel implant devices, the preoperative radiographic assessment of the cochlear architecture has become more critical.

The modalities of imaging that are most pertinent to evaluation of auditory pathway are high resolution computed tomography (HRCT) and magnetic resonance imaging (MRI) scans.

Preoperative imaging often provides valuable information that would not preclude implantation but rather helps assessing in which ear it would be technically easier or better to implant a device [5].

The preoperative sectional imaging may derive additional useful information that can optimize safety and facilitate surgery, as well as influencing subsequent patient management. Proper surgical planning must involve careful review of sectional images, so that potential complications may be anticipated and properly managed [6].

There is a paucity of literature from India and for this reason we decided to conduct this study in Indian context.

*Aim of the Study*. To find out various congenital inner ear malformations by radiological assessment in a tertiary care centre.

## 2. Material and Methods

This prospective analytical study was undertaken in the Department of Otorhinolaryngology and Head and Neck Surgery at Sir Ganga Ram Hospital (SGRH), New Delhi, from January 2003 to June 2013. We evaluated a total of 280 children (males: 158, females: 122) of age of 01–14 years with standard deviation (SD) of 2.8171 and mean age 2.76 years, with bilateral congenital severe to profound sensorineural hearing loss. All patients had congenital deafness and showed bilateral severe to profound sensorineural hearing loss in observational audiometric tests, otoacoustic emissions, and auditory brain stem responses. All patients were candidates for possible cochlear implantation; the patients underwent HRCT and MRI examination of the temporal bone and inner ear. To reduce motion artifacts, the children were studied in sedation. The patients included in the study were selected on the basis of following inclusion and exclusion criteria.

 Inclusion criteria: children who were congenitally deaf.
 Exclusion criteria: children who were not congenitally deaf and developed hearing loss after some acquired cause.

Work-up of the patient includes the brief history of the patient which includes history of hearing loss, prenatal, natal, and postnatal history, drug intake and radiation exposure to mother during pregnancy, developmental history, hearing aid trial, any other associated diseases, and family history.

Examination of patient includes the general examination, ear, nose, and throat examination, and any syndromic signs.

Investigations includes the audiological assessment of patient which includes pure tone audiogram (PTA), free field audiometry (FFA), brain evoked response audiometry (BERA), auditory steady state response (ASSR), and otoacoustic emissions (OAE).

Radiological assessment by HRCT temporal bone and MRI head with 3D reconstruction of cochleovestibular complex to see the status of

- morphology of cochlea with modiolus,
- vestibule,
- vestibular aqueduct,
- semicircular canals,
- internal auditory canal,
- status of vestibulocochlear nerve.

### 2.1. Imaging Protocol

#### 2.1.1. HRCT Scan

All HRCT investigations were performed in the axial orientation using multislice light speed with a slice thickness of 0.625 mm and ultrahigh algorithm. These were documented in a bone window. Coronal and sagittal reconstructions were performed with volume rendered images if required. All images were evaluated as advantage windows work stations.

#### 2.1.2. MRI Scan with 3D Reconstruction of Cochlea

All MRI scans were performed on a 3T MRI scanner (Siemens Verio) using an 8-channel head coil and the SPACE (heavily T2 weighted) sequence. Images were viewed on a Siemens work station in multiple planes with MIP and 3D reconstruction.

#### 2.1.3. Sedation

In some of the children up to age of 4 years, Triclofos (5 mL = 750 mg) was given in the dose of 50–70 mg/kg of body weight. For elderly children of age group >4 years, midazolam (0.05–0.1 mg/kg of body weight) was given intravenous.

#### 2.1.4. Image Analysis

All printed CT and MRI were evaluated independently by a senior ENT surgeon, a senior radiologist, and ENT resident. Different parts of inner ear were studied for malformations. The morphology of the cochlea, vestibule, semicircular canals, vestibular aqueduct, and internal auditory canal along with vestibulocochlear and facial nerve is described. The malformations were classified using new classification of inner ear malformations based on CT and MRI given by Sennaroglu and Saatci [6]. The evaluation of nerves within the internal auditory canal was performed with the reconstructed axial and parasagittal MR images. The complete course, from the brain stem into the labyrinth, of the nerves was studied. A present facial nerve and vestibulocochlear nerve branching into the cochlear, inferior, and superior vestibular nerve were identified as normal.

Data was collected and entered in a predesigned proforma which includes patient's demography, patient's clinical work-up, audiological findings, and radiological findings, and the results were analyzed.

Radiological findings were arranged as per classification given by Sennaroglu and Saatci [6].

## 3. Results and Analysis

HRCT and MRI depicted numerous congenital malformations of the inner ear. There was no difference in describing anomalies of the inner ear between both modalities. CT allowed appreciation of the bony borders of the malformations, and MRI showed the fluid filled cavities.

A total of 280 children (560 ears) with the age group of 01 to 14 years with bilateral congenital severe to profound sensorineural hearing loss were radiologically evaluated with HRCT of temporal bone and MRI of inner ear. Out of 280 children, 240 children were normal and 40 children (78 inner ears) were found to be congenital abnormal. All the 40 children had bilaterally abnormal inner ear except for 2 children who had unilateral abnormal ear.

## 4. Evaluation of the Anomalies

### 4.1. Cochlear Anomalies

Out of 78 abnormal inner ears, in 57 (73%) cochlea was found to be abnormal. Abnormalities of cochlea includes the incomplete partition type-I (IP-I), incomplete partition type-II (IP-II), and common cavity deformity.

In 9 (11.5%) inner ears, cochlea had no turn or only a bony mass without any turn was visualized so it was classified as incomplete partition type-I (IP-I).

In 32 (41%) inner ears, cochlea was of incomplete partition type-II (IP-II), means Mondini deformity, in this type the cochlea consists of 1.5 turns in which the middle and apical turns coalesce to form a cystic apex, accompanied by a dilated vestibule and enlarged vestibular aqueduct.

In 16 (20.5%) of cases cochlea was classified under the common cavity as there was cystic cavity representing the cochlea and vestibule, without showing any differentiation into cochlea and vestibule.

**Figure 1:**
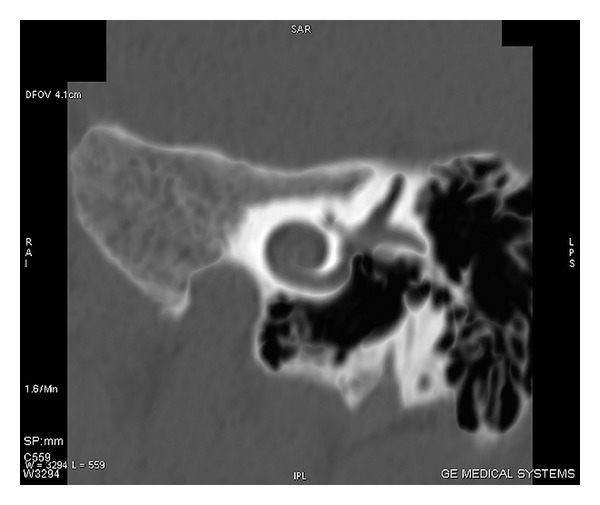
HRCT scan of temporal bone with coronal section showing cochlear anomaly in which cochlea shows 1 and 1/2 turns.

**Figure 2:**
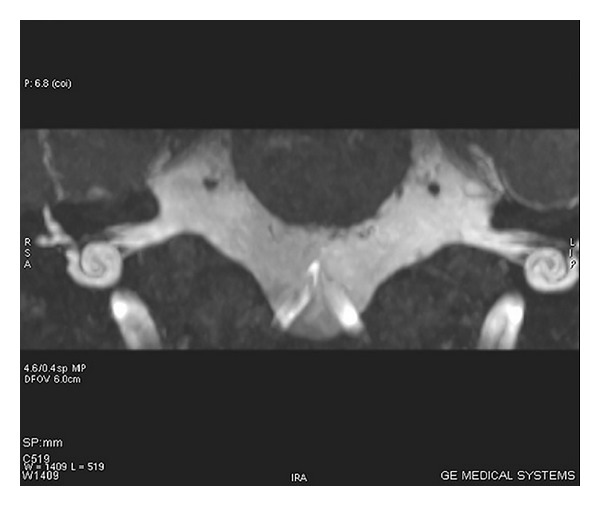
MRI scan of inner ear with axial section showing cochlear anomaly in which cochlea shows 1 and 1/2 turns.

**Figure 3:**
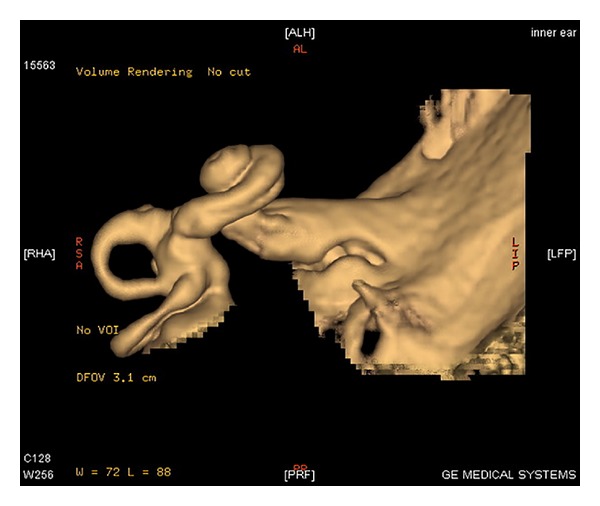
MRI scan with 3D reconstruction of inner ear showing cochlear anomaly in which cochlea shows 1 and 1/2 turns.

Modiolus was absent in 25 (32%) inner ears and in the rest of the cases it was normal (Figures 1, 2, and 3).

#### 4.1.1. Vestibular Anomalies

Vestibular anomalies were the most common anomalies found. Out of 78 abnormal inner ears in 68 (87.1%) inner ears vestibule was found abnormal. In 62 (79.4%) inner ears vestibule was dilated and in the rest 6 (7.6%) it was aplastic or hypoplastic.

#### 4.1.2. Semicircular Canal Anomalies

In 21 (26.9%) out of 78 malformed inner ears, lateral semicircular canals were found to be aplastic or hypoplastic and in 10 (12.8%) inner ears lateral semicircular canal was dilated. Superior semicircular canal was aplastic or hypoplastic in 11 (14.1%) cases and dilated in 3 (3.8%) cases. Posterior semicircular canal was found to aplastic or hypoplastic in 14 (17.9%) inner ears and was dilated in 3 (3.8%) cases. In 7 (8.9%) cases all the three canals were absent and in 3 (3.8%) cases all the three canals were dilated.

#### 4.1.3. Vestibular Aqueduct Anomalies

In 44 out of 78 (56.4%) of abnormal inner ears the vestibular aqueduct was found to be abnormal. Vestibular aqueduct was found to be dilated in 41 (52.5%) of cases and in the rest 3 (3.8%) of cases it was aplastic or hypoplastic.

#### 4.1.4. Internal Auditory Canal (IAC) Anomalies

In 24 out of 78 (30.7%) of abnormal inner ears the internal auditory canal was found to be abnormal.

**Figure 4:**
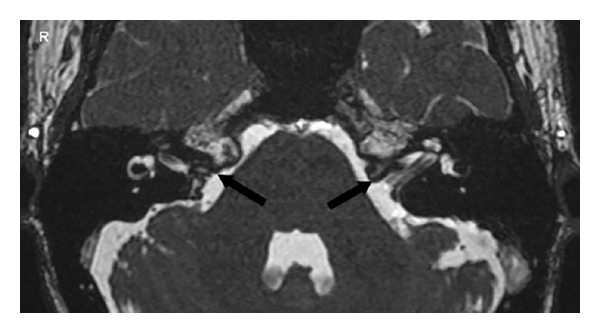
MRI scan of inner ear with axial section showing bilateral hypoplastic internal auditory canals.

In 19 (24.3%) inner ears IAC was found to be narrow in lumen and short in length. In most of the cases its diameter was <2 mm and short in length also. In 2 (2.5%) cases it was absent and only a solid bony structure which was not patent was seen. In 3 (3.8%) cases IAC was dilated with the diameter of >4 mm (Figure 4).

#### 4.1.5. Status of Vestibulocochlear Nerves

**Figure 5:**
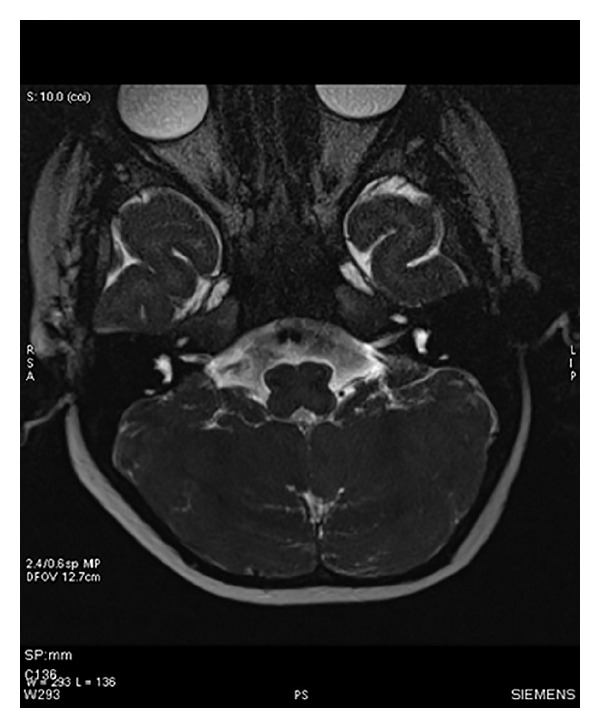
MRI scan of inner ear with axial section showing bilateral hypoplastic vestibulocochlear nerves.

**Figure 6:**
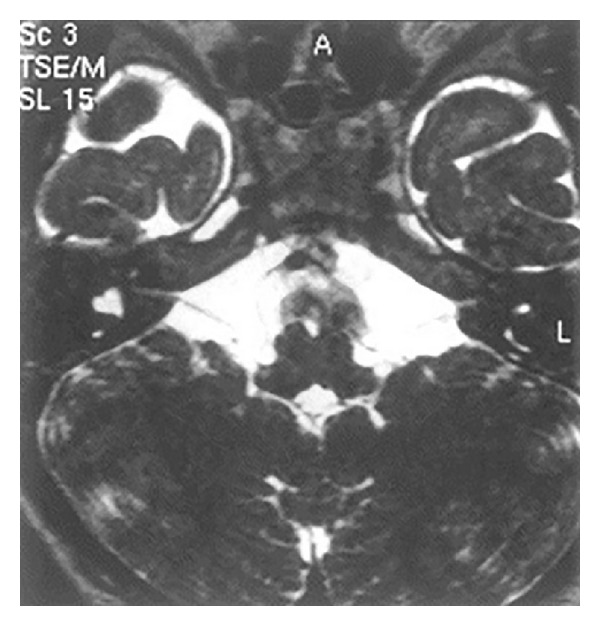
MRI scan of inner ear with axial section showing bilateral hypoplastic vestibulocochlear nerves.

In all cases where IAC was malformed, vestibulocochlear nerves were also malformed except for 1 case where IAC was dilated but nerves were visualized. Out of 78 inner ears, 23 (29.4%) inner ears had nerve anomalies. In 11 (14.1%) of cases nerves were thin in diameter but well visualized; in 12 (15.3%) cases nerves were absent or not visualized (Figures 5 and 6).

## 5. Overall Evaluation of the Malformations

A total of 313 malformations were detected in 78 abnormal inner ears in a total of 40 patients. 57 of 313 (18.2%) inner ear malformations showed malformations of cochlea and in 25 of 313 (7.9%) inner ear malformations modiolus was found to be malformed. In 44 of 313 (14%) inner ear malformations vestibular aqueduct was abnormal. In 68 of 313 (21.7%) inner ear malformations vestibule was abnormal. In 72 of 313 (23%) semicircular canals were found to be malformed. In 24 of 313 (7.6%) inner ear malformations internal auditory canal was found to be malformed. In 23 of 313 (7.3%) vestibulocochlear nerves anomalies were present.

Summary is shown in Table 1.

Maximum malformations found in a single ear were 7 structural malformations which included malformation of cochlea, modiolus, vestibule, vestibular aqueduct, semicircular canals, internal auditory canal, and vestibulocochlear nerve. Five out of 78 (6.4%) malformed inner ears showed all 7 structural malformations, 6 ears (7.6%) had 6 malformations, 7 ears (8.9%) had 5 malformations, 15 ears (19.2%) showed 4 malformations, 19 ears (24.3%) had 3 malformations, 23 ears (29.4%) had 2 malformations, and only 3 ears (3.8%) had single isolated malformation.

Summary is given in Table 2.

## 6. Discussion

In present study, we identified total number of 313 inner ear malformations in 78 inner ears in a total of 40 patients with HRCT scan and MRI scan. This study showed that HRCT scan and MRI scan revealed similar morphologic findings of malformed inner ears, except for vestibulocochlear nerves which were more appreciated on MRI scan. The importance of HRCT scan to study the temporal bone should not be underestimated [7–9]. HRCT scan depicts the bony borders of malformed labyrinth. This is important because the surgeon can analyze the direction of insertion of the electrode array to minimize the risk of misplacement and by assessing the malformation preoperatively we can minimize the trauma to the vital structure. The implantation of the cochlear implant requires the knowledge about the cochleovestibular malformations. MRI scan delivers additional information that is needed in the preoperative work-up of patients with congenital sensorineural deafness. The fluid filled spaces of the normal cochlea and the malformed cochlea are necessary for the insertion of the electrode array of the cochlear implantation [10]. This can be clearly visualized with MRI scan by using a 3D T-2-weighted fast SE sequence for the surgical reasons and for proper evaluation of congenital malformations only the combined use of HRCT scan and MRI scan can be recommended to study this patient group.

One of the most important findings of our study is that MRI scan allows full appreciation of the normal anatomy and anomalies of the vestibulocochlear nerves within the internal auditory canal in children with congenital sensorineural deafness. For this we performed modified acquisitions of axial and parasagittal reformations using small field of view. In 23 inner ears MRI documented anomalies of vestibulocochlear nerves within the internal auditory canal. Clinical significance of these findings is important. A missing or ill-defined vestibulocochlear nerve is a contra indication for cochlear implantation surgery because this nerve is required to conduct the cochlear implant impulses [11–13].

In the clinical setting, evoked potentials may be used to study the presence and function of the nerve. A positive brain stem evoked potential predicts a functional nerve, but a negative test does not distinguish between a functional, damaged, or undeveloped nerve [13]. 11 inner ears were found to have bilateral ill-defined but visualized vestibulocochlear nerves and 12 inner ears had absent nerves. MRI scan detected anomaly of the vestibulocochlear nerve in 23 inner ears out of 78 (29.4%) malformed ears in this study population but in study by McClay et al. in 2008 [14] and Miyasaka et al. in 2010 [15] it found 40% and 18% of nerve anomalies, respectively. In the present study the internal auditory canal was malformed in 24 out of 78 (30.7%) abnormal inner ears; in 19 (24.3%) inner ears IAC was found to be narrow in lumen and short in length. In most of the cases its diameter was <2 mm and short in length also and this was associated with absent or thin nerves, in 2 (2.5%) cases it was absent and only a solid bony structure which was not patent was seen, and in 3 (3.8%) cases IAC was dilated with the diameter of >4 mm. Westerhof et al. in 2001 [16] found 38% of internal auditory canal anomalies in their study which is slightly higher than present study.

Anomaly of the vestibulocochlear nerve occurred along with a malformed labyrinth. Data from embryologic studies might explain this phenomenon. In the ninth embryonic week, the cochlear windings are developed and the rise of neural epithelium builds a cochlear ganglion and neural fibers (early cochlear nerve) start to develop. These fibers grow centrally to the brain stem and peripherally back into the otic epithelium. Initial afferent fibers entering the undifferentiated otic epithelium are appreciated in the 10th embryonic week [17]. A nerve growth factor like substance released by the otic vesicle which is essential for the survival of the neural cell supports this development [18]. These data explain that in case of an arrest in the developing labyrinth, the neural embryonic proceedings may be disturbed. This could result in anomaly or aplasia of the vestibulocochlear nerves, which we found in 29.4% of ears with anomaly of the bony labyrinth.

Our study illustrates that imaging studies in patients with congenitally sensorineural hearing loss should not focus just on the vestibulocochlear nerves [19] or on cochlea [20, 21]. The majority of our patients demonstrate multiple anomalies of the inner ear. We have classified the anomalies according to the latest classification of congenital inner ear malformations given by Sennaroglu and Saatci in 2002 [6].

### 6.1. Incomplete Partition Type-I (IP-I)

Incomplete partition type-I (cystic cochleovestibular malformations) is a malformation involving the cochlea and vestibule. In a case of IP-I, a cystic dilated vestibule accompanied the cystic, empty cochlea. This pathology represents a form of common cavity that is one step more organized and differentiated than common cavity [6]. In our study 9 (11.5%) inner ears were classified under this category, in these cases the dimensions of the cochlea were normal but the internal architecture was missing, and there was no modiolus in the cochlea giving it the shape of an empty cystic structure. Vestibule was grossly enlarged and the vestibular aqueduct was also dilated. The studies conducted by Sennaroglu and Saatci in 2002 [6] and Westerhof et al. in 2001 [16] found the incidence of IP-I as 8% and 12%, respectively, and this is almost similar to our results. The arrest of development should be at the 5th week. In addition the histological presentation of the patient reported by Graham et al. [22] fits IP-I because there are two separate cavities, although they described it as common cavity. The case presented as common cavity by Swartz and Harnsberger in their radiology text book also has separate cystic cochlear and vestibular components and is, we think, another example of IP-I but we have classified these cases separately as common cavity, in which cochlea was classified under the common cavity as there was cystic cavity representing the cochlea and vestibule, without showing any differentiation into cochlea and vestibule and the incidence of these type of cases in our study was 16 (20.5%) as compared to 7% of study by the Sennaroglu and Saatci [6].

### 6.2. Incomplete Partition Type-2 (IP-2): Mondini Malformation

The malformation of incomplete partition type II (Mondini malformation) represents cochlea in which only the basal part of the modiolus is present. This is the type of cochlea originally described by Carlo Mondini and together with a minimally dilated vestibule and large vestibular aqueduct it constitutes the triad of the Mondini deformity. This gives the apex of the cochlea a cystic appearance due to the confluence of the middle and apical turns. In our study, out of 78, 32 (41%) inner ears were classified in incomplete partition type-II according to Sennaroglu and Saatci classification. In these 32 inner ears cochlea was malformed having 1 and 1/2 turns with normal modiolus, dilated vestibule along with dilated vestibular aqueduct. In the studies conducted by Sennaroglu and Saatci in 2002 [6] and Westerhof et al. in 2001 [16] they found the incidence of IP-II was 15% and 22%, respectively. It is thought that in these types of malformations the arrest of development is at the 7th week of gestation.

In the present study we found total 14.2% of children with vestibulocochlear anomalies which is lower incidence in comparison to the international studies, and according to them the incidence is about 20% (Sennaroglu and Saatciin 2001 [6]), 23% (Abdullah et al. in 2003 [23]), 30% (Ma et al. in 2008 [24]) and 31% (McClay et al. in 2008 [14]). All of these studies had lower sample size than our study.

## 7. Conclusion 

The present study is the first study done in India. By this study we can find out different types of congenital inner ear malformations and their incidence in congenitally deaf children in India.

## Figures and Tables

**Table 1:** Overall evaluation of malformations.

Type	Number	Percent (%)
Cochlear	57/313	18.2%
Modiolus-	25/313	7.9%
Vestibular aqueduct	44/313	14%
Vestibule	68/313	21.7%
Semicircular canal	72/313	23%
Internal auditory canal	24/313	7.6%
Vestibulocochlear nerve-	23/313	7.3%

**Table 2:** Distribution of malformations.

Number of malformations	Number. of inner Ears	Percent (%)
Seven	5/78	6.4%
Six	6/78	7.6%
Five	7/78	8.9%
Four	15/78	19.2%
Three	19/78	24.3%
Two	23/78	29.4%
One	3/78	3.8%
